# Adherence tool for prophylactic haemophilia treatment in adult and adolescent patients: A systematic review and meta-analysis protocol

**DOI:** 10.1371/journal.pone.0289815

**Published:** 2023-12-14

**Authors:** Fadzlin Mohd Mokhtar, Sutha Rajakumar, Hasniza Zaman Huri

**Affiliations:** 1 Department of Clinical Pharmacy and Pharmacy Practice, Faculty of Pharmacy, University Malaya, Kuala Lumpur, Malaysia; 2 Jeffery Cheah School of Medicine and Health Sciences, Monash University Malaysia, Subang Jaya, Malaysia; Universite de Nantes, FRANCE

## Abstract

Hemophilia is a congenital bleeding disorder resulting from a low level or deficiency of clotting factors. It is an x-linked recessive disease and happens almost exclusively in males whereas females are the carrier of the affected gene. The most common types of hemophilia are hemophilia A and Hemophilia B. Hemophilia is classified into mild, moderate and severe. Prophylaxis treatment has more advantages clinically compare to on-demand therapy. It may reduce the bleeding frequency, gives protection from joint damage, may lower the number of total bleeding episodes per year, and may reduce annualised spontaneous and trauma related bleeding events. However, prophylaxis treatment needs regular weekly infusions therefore it is painful to administer especially if the vein is difficult to access. It may cause pain at the site of injections and may lead to non-adherence to treatment. Non-adherence to a regimen will result in insufficient clotting factor levels in the body. The efficacy of the medication is reduced and may lead to a high bleeding tendency. Thus far, the study on adult haemophilic patient adherence tool is scarce and limited; and therefore this review is warranted. The study protocol is conducted as per the PRISMA-P guideline. There are 4 concepts in this systematic review which are Haemophilia, adult and adolescence, preventive treatment and adherence. Articles will be sought from electronic databases PUBMED, Ovid EMBASE, CINAHL, and SCOPUS using the MeSH term, synonym free-text word, truncation, and proximity operators as per each database. The proposed keywords within each concept will be joined using the Boolean operator “OR “and the 4 different concepts combined using the Boolean operator “AND”. Search will be limited to Human, English language, and publication until 2022. Studies will be included if they meet the study inclusion criteria. The quality of the studies will be appraised using the Newcastle-Ottawa quality assessment scale (NOS) for observation-based studies. This systematic review does not require formal ethical approval as data will be extracted from selected published studies. The results will be disseminated through a peer-reviewed publication and relevant conference presentations.**(**PROSPERO registration CRD42021273813)

## Introduction

Hemophilia is a congenital bleeding disorder resulting from a low level or deficiency of clotting factors. It is an x-linked recessive disease and happens almost exclusively in males whereas females are the carrier of the affected gene. According to the World Federation of Hemophilia’s (WFH) Annual Global Surveys 2017, a total of 158,225 people have been identified as haemophilia A based on 116 reporting countries which represent 91% of the world population [1]. One case of haemophilia A occurs per 5000 males worldwide, and one-third of those infected do not have a family history of the condition. Haemophilia A is more common in some countries than others, with 5.4 to 14.5 cases per 100,000 males. [2]. According to estimates, the cost of treating severe haemophilia in European nations totalled EUR 1.4 billion in 2014, or slightly under EUR 200,000 per patient. With a study average of EUR 199,541, Germany had the greatest per-patient expenses (mean EUR 319,024) and the lowest (mean EUR 129,365).As expected, clotting factor replacement therapy consumption represented the vast majority of costs up to 99% [3].

The most common types of hemophilia are hemophilia A and Hemophilia B. Hemophilia is classified into mild, moderate and severe. Severe hemophilia patients (FVIII:C or FIX:C levels 5–20% of normal) would present with spontaneous bleeding episodes without a history of trauma. Patients with severe hemophilia will be on prophylaxis treatment as a gold standard to ensure the level of clotting factor at 1% or higher at all times [4]. Prophylactic treatment of haemophilia is defined as regular administration of clotting factor concentrates for more than two months to prevent bleeding episodes [5]. Benefits of prophylaxis include decreased frequency of bleeding episodes, emergency room visits and hospitalizations [6]. Prophylaxis treatment has more advantages clinically compared to on-demand emergency room visits and hospitalizations [6]. Prophylaxis treatment has more clinical advantages than on-demand therapy. However, prophylaxis treatment needs regular weekly infusions therefore it is painful to administer especially if the vein is difficult to access. It may cause pain at the site of injections and lead to non-adherence to treatment. Severe haemophilia due to non-adherence is associated with a major psychological and economic burden for patients, caregivers, and the wider health care system. According to the World Federation of Haemophilia 2012 guidelines, there are three major complications in haemophilia. There are musculoskeletal complications, the development of inhibitors and transfusion-transmitted and other infection-related complications.

Several studies have measured adherence to prophylaxis [7–10]. Measures of treatment adherence also vary widely and may include data from treatment logs, pharmacy records, bleed frequency including joint bleeds [11–14]. These tools consist of surveys using validated self-constructed questionnaires or a validated Validated Hemophilia Regimen Treatment Adherence Scale(VERITAS) questionnaire, pharmacy logs, as well as infusion log [15, 16]. For surveys, the scoring of questionnaires was used to determine the adherence level. As for pharmacy and infusion logs, measurements focus on drug use and intake percentage. In this study, Veritas questionnaires were used which could be easily applied to achieve high response rates. It was pioneered in United States and has been widely used since 2010 [17–20]. All methods can be used as no gold standard has been developed for evaluating medication adherence [21]. Non-adherence to the regimen will result in insufficient clotting factor levels in the body. The efficacy of the medication is reduced and may lead to a high bleeding tendency. Thus far, the study on adult haemophilic patients is scarce and limited and therefore this review is needed to provide insight of treatment adherence. By identifying poor adherence patients (through tools of adherence used) intervention can be made to increase adherence which may avoid severe complications. The following research questions were explored with this protocol:-”1. To determine the most frequently reported areas and common tools used for adherence measurement in Haemophilia; 2. To make recommendations on the choice of the adherence instruments for Haemophilia.”

## Methods and design

### Selection criteria

This protocol for this study was developed based on the PRISMA-P guidelines. The eligibility criteria for the selection of studies are related directly to the research question and will determine the selection of the studies included in the systematic review and meta-analysis. The eligibility criteria set in terms of inclusion and exclusion criteria will enable reviewers to limit bias. Setting an eligibility criterion validates and facilitates the researcher’s screening process. The inclusion and exclusion criteria for this systematic and meta-analysis are as follows:

### Inclusion

Any study design

Population: Adult and adolescents with Haemophilia

Intervention/Exposure: preventive or prophylaxis treatment

Outcome: adherence

Papers would also be required to fulfil the following inclusion criteria other than study design & PEO:

1.Original articles published in the English language only

2.Human studies

3.Original research published in peer-reviewed scientific journals

### Exclusion

This study will exclude the following:

Studies conducted on animals.

Genetic based studies

### Public involvement

The study does not include active recruiting. Data will be extracted from published articles of selected peer-reviewed journals.

### Search strategy

This systematic review will be conducted by searching electronic databases such as PUBMED, Ovid EMBASE, CINAHL, and SCOPUS. Manual hand search would also be conducted in the references of the selected studies to identify studies adhering to the inclusion criteria. The assistance of the university librarian will be sought during the designing of the search strategy and the final search strategy will be double-checked by authors FMM, SR, and HZH.

The proposed keywords in the preliminary search are as follows:

Concept 1 Haemophilia

Concept 2 adult and adolescence

Concept 3 preventive treatment OR prophylaxis

Concept 4 adherence

**Fig 1:**
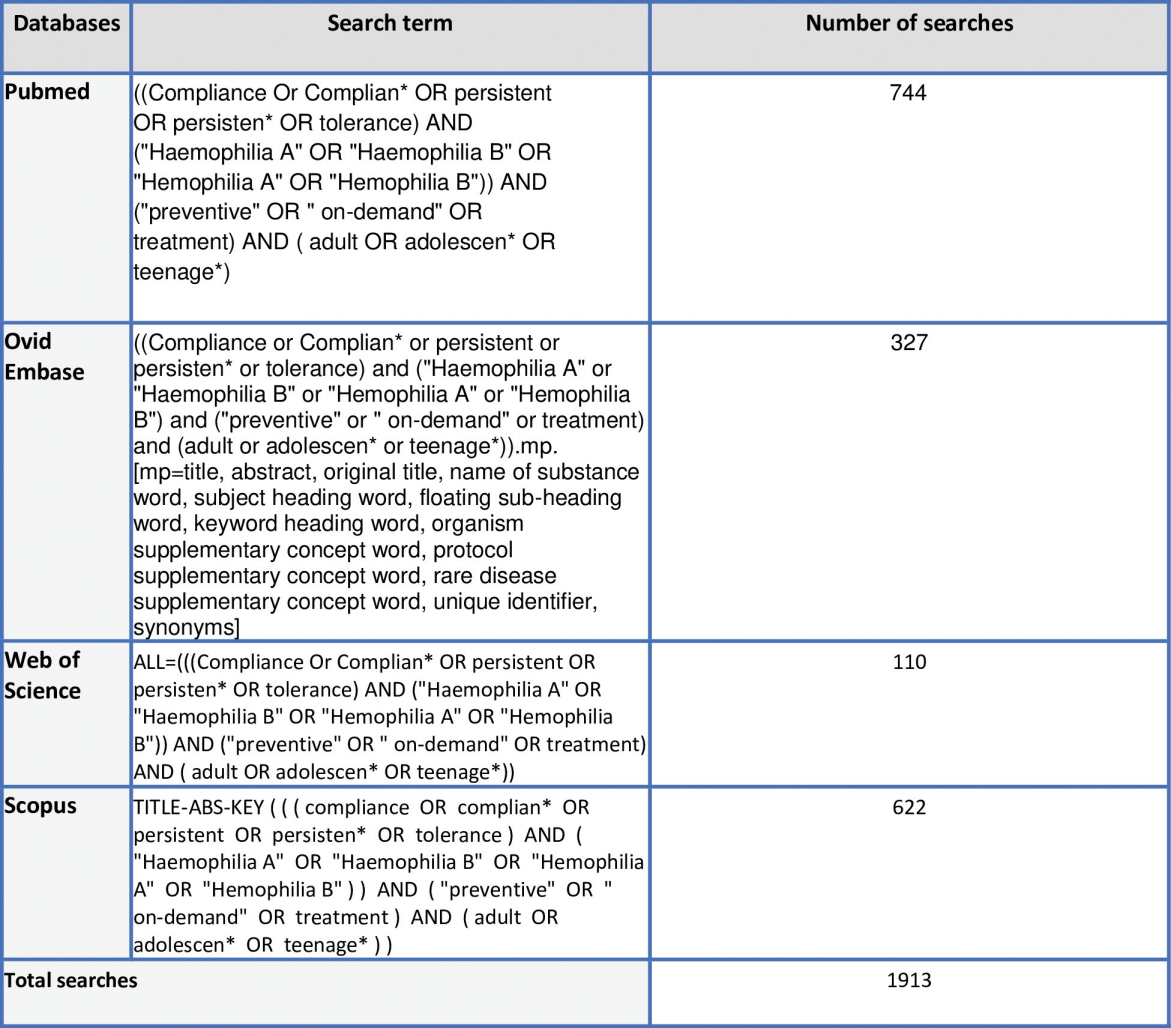
Database searches and preliminary search results.

Eligible studies will be searched using the MeSH term, synonym free-text word, truncation, and proximity operators as per each database. The proposed keywords within each concept will be joined using Boolean operator “OR “and the 4 different concepts will be combined using the Boolean operator “AND”(Fig 1). Search will be limited to Human, English language, and publication from the year 2000 to 2020 for contemporary evidence. The literature search will be carried out in 4 databases which are PubMed, Ovid EMBASE, CINAHL, and SCOPUS (Fig 1). The different databases were used to search to minimize selection bias. Refer to Fig 1 for the samples of the proposed search strategy for each database. The references of the selected studies will undergo a manual hand search to identify more studies. Grey literature such as abstract conferences will also be considered if sufficient information is present. All the search results will be imported to Endnote and merged. Upon merging it will be transferred to Covidence, a systematic review software. The software facilitates in removing duplicates, screening, and data extraction process.

### Selection of studies

After the articles have been identified from the 4 databases and duplicates being removed, two authors (FMM & SR) will conduct screening of the titles and abstracts in Covidence according to the inclusion and exclusion criteria. Any conflicts will be resolved by the reviewer (SR) with a vote of “YES” or “NO” after discussion with senior researcher (HZH). The selected articles through an electronic database and manual hand search of reference of selected studies will undergo full-text review according to the inclusion and exclusion criteria by the review author (FMM & SR). Any arising difficulty will be discussed with senior researcher (HZH). The study will be conducted per the Preferred Reporting Items for Systematic Reviews and Meta-Analyses Protocols (PRISMA) checklist. An auto-generated PRISMA flow diagram would be obtained from Covidence software to elucidate the selection process.

### Quality assessment

Risk of bias assessment will be done by two reviewer authors (FMM & SR) using a suitable assessment tool. Any queries arising during the risk of bias assessment will be discussed and resolved with review author HZH. Each study will be checked for any bias based upon the different components specified in the tool. The studies analysed would not be randomised therefore Newcastle Ottawa scale (NOS) would be used. Newcastle Ottawa Scale is broken down to 3 types to cover for cross-sectional, cohort and case-control studies. For observational studies, an increased number of stars allocated to the domains of selection, comparability, and outcome/exposure, with higher scores is of better quality [22].

### Data extraction and management

Review author (SR) will export selected studies after the full-text review in Covidence software to Microsoft Excel. Data will be extracted as per the review questions and fulfil the narrative synthesis conditions (HZH &SR). Review authors HZH and SR will double-check the extracted data, and all conflicts will be resolved.

### Analysis (Data synthesis)

#### Narrative synthesis

A narrative synthesis will be done using data from the included studies after the final screening. Four tables will be created as follows to record:

1) Characteristics of the included studies

2) Methodology, including adherence tool used to determine adherence and their validity and reliability;

3) Covariables in the observational studies included as confounding variables are the major problem for observational studies;

The narrative synthesis will describe the similarities and differences between the findings. The findings’ strengths and weaknesses, such as the influences of confounding variables, will also be discussed.

#### Heterogeneity and meta-analysis

Studies in the narrative synthesis will undergo heterogeneity assessment for inclusion in the meta-analysis. Higgins et al argued that statistical heterogeneity is inevitable since clinical and methodological diversity always occurs in a meta-analysis. However, methods such as I^2^ statistics have been developed to quantify inconsistency among studies to assess its impact on the meta-analysis. The I^2^ statistic will be used to assess heterogeneity across studies with cut-off values: -Mild heterogeneity 25–50%, Moderate heterogeneity 50–75%, and Severe heterogeneity >75% [23]. Publication bias will be evaluated by visually examining the funnel plot. Suitable statistical analyses such as odd ratio will be performed to determine the odds ratio of different adherence tools. The analysis will be done at a 95% confidence interval. If there is presence of 2 or more studies report output or information from the same data, the analysis will be conducted on the study with the most participants.

## Conclusion

This systematic review will be the first of its kind to use various research work to draw up conclusions surrounding adherence with various adherence tools used in reporting prophylactic treatment.
